# High-Throughput Determination of Sodium Danshensu in Beagle Dogs by the LCMS/MS Method, Employing Liquid-Liquid Extraction Based on 96-Well Format Plates

**DOI:** 10.3390/molecules22050667

**Published:** 2017-04-25

**Authors:** Jingjing Jiang, Xin Zhao, Xiuxiu Li, Shengyuan Wu, Shidan Yu, Yuefen Lou, Guorong Fan

**Affiliations:** 1 Shanghai Key Laboratory for Pharmaceutical Metabolite Research, School of Pharmacy, Second Military Medical University, Shanghai 200433, China; jiangjingj8@163.com (J.J.); Lixiuxiu0217@163.com (X.L.); woshiysd@163.com (S.Y.); 2 Department of Pharmaceutical Analysis, School of Pharmacy, China Pharmaceutical University, No. 24 Tong Jia Xiang, Nanjing 210009, China; yoyo0132@163.com; 3 Laboratory of Drug Metabolism & Pharmacokinetics, School of Medicine, Tongji University, No. 1239 Siping Road, Shanghai 200092, China; wu-shengyuan@tongji.edu.cn; 4 Department of Pharmacy, Branch of Shanghai First People’s Hospital, Shanghai 200081, China; 5 Department of Clinical Pharmacy, Shanghai General Hospital, School of Medicine, Shanghai Jiao Tong University, No. 100 Haining Road, Shanghai 200080, China

**Keywords:** Sodium Danshensu, pharmacokinetics, LC-MS/MS, 96-well liquid-liquid extraction, Beagle dogs

## Abstract

Sodium Danshensu (sodium d-(+)-β-(3,4-dihydroxyphenyl) lactate), one of the water-soluble ingredients in *Salvia miltiorrhiza*, exhibits potent relaxation of the coronary artery and anticoagulation effection. A high-throughput, rapid, and sensitive method combining liquid chromatography with electrospray ionization tandem mass spectrometry to determine the sodium danshensu in beagle dog plasma was developed and validated, using gallic acid as an internal standard (IS). Acidified plasma samples were extracted using 96-well liquid-liquid extraction, and were eluted on a CNW Athena C18 column (3 μm, 2.1 × 100 mm) by using a gradient mobile phase system of methanol and water (containing 0.2% formic acid). The mass spectrometric detection was achieved using negative ion electrospray ionization mode and monitoring the precursor→production combinations of *m*/*z* 197→135 for sodium danshensu and 169→125 for IS, in multiple reaction monitoring modes. Good linearity was achieved, and the linear range was 10–1000 ng/mL (R^2^ > 0.996) with a quantification limit of 10 ng/mL for sodium danshensu in beagle dog plasma. The intra- and inter-day precision (RSD) ranged from 2.1% to 9.0%. The accuracy (RE) was between −8.6% and 5.7% at all quality control levels. The validated method was successfully applied to the pharmacokinetics study of sodium danshensu in beagle dog plasma after intravenous injection and oral administration of sodium danshensu.

## 1. Introduction

**Figure 1:**
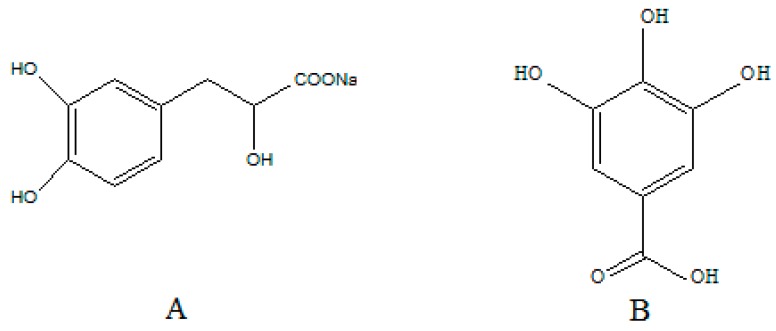
Chemical structures of sodium danshensu (**A**) and gallic acid (IS, **B**).

Sodium danshensu (sodium d-(+)-β-(3,4-dihydroxyphenyl) lactate) is a sodium salt of danshensu extracted from Danshen (Figure 1). As a popular traditional Chinese medicine, Danshen is the dried root of the traditional Chinese herb *Salvia miltiorrhiza* that belongs to the family of *Labiatae*, and has been used extensively in many traditional Chinese medicine prescriptions for treating various diseases, especially in cases of hepatitis, and cerebrovascular and cardiovascular diseases [1], such as cardiodynia, myocardial infarction [2], intimal hyperplasia, and attenuated restenosis [3]. According to the study of phytochemistry, the main bioactive constituents in the roots of Danshen can be classified into two broad categories: the hydrophilic phenolic acid compounds and the lipophilic tanshinone compounds [4,5]. Among these constituents, danshensu is one of the major constituents of hydrophilic phenolic acid compounds, which has been shown to exert several pharmacologic effects such as a cardioprotective effect [6], prevention of ischemia/reperfusion injury [7,8], anti-oxidative damage [9], hydroxyl radical scavenging roles [10], and so on. Therefore, sodium danshensu as a candidate compound has attracted considerable attention, and it is necessary to study the pharmacodynamics, pharmacokinetics, and safety of sodium danshensu.

Excellent pharmacokinetic properties are significant criteria for assessing the drug-likeness of candidates. In order to investigate the pharmacokinetics of sodium danshensu, several methods have been reported for its determination in biological matrixes, such as liquid chromatography coupled to fluorescence [11], HPLC-UV [12,13,14], or HPLC-MS/MS [15,16,17,18,19,20,21,22]. HPLC-UV and fluorescence, the common analytical methods, possess major drawbacks of low-concentration sensitivity and being time-consuming, which cannot be ignored. Since the combination of powerful separation from HPLC and the superior selectivity and sensitivity from mass spectrometry made liquid chromatography-tandem mass spectrometry (HPLC-MS/MS) one of the most useful techniques in bioanalytical chemistry, HPLC-MS/MS has been used as a prevalent method for analyzing sodium danshensu in pharmacokinetic studies. However, among these studies, a majority of them investigated the concentration of sodium danshensu in human or animals after administering Danshen extracts or compound preparations such as dripping pill, tablet, or capsule [23,24,25,26], meaning that the other components in the drugs may affect the absorption and distribution of sodium danshensu, and then affect the determination and pharmacokinetic study. Therefore, in order to better explain its pharmacokinetic characteristics, it is necessary to study the sodium danshensu solely. In this study, a high-throughput, simple, sensitive, selective, and reliable LC-MS/MS method was developed and validated for the pharmacokinetic and bioavailability studies of sodium danshensu in beagle dog plasma after single intravenous injection doses at 5, 10, and 15 mg/kg and an oral dose at 60 mg/kg. The data presented in this study provides useful information for the further study of the druggability of sodium danshensu.

## 2. Results and Discussion

### 2.1. Selection of Sample Preparation and Internal Standard

Classic pretreatment methods include protein precipitation, liquid-liquid extraction, and solid-phase extraction. In the preliminary experiment, solid phase extraction is abandoned. On the one hand, the retention capacity of the water-soluble sodium danshensu on the solid-phase extraction cartridge is weak, resulting in the difficulty of selecting a solid phase extraction column. On the other hand, the use of solid phase extraction is more time-consuming and expensive. To establish a sample preparation procedure with high efficiency and improved recovery of the analyte and IS, different methods including protein precipitation and liquid-liquid extraction (LLE) in the 96-well plate format were compared. Protein precipitation, as the simple and fast method, was attempted for the sample preparation initially. The result demonstrated that the protein precipitation using methanol or acetonitrile as the extraction solvent resulted in low extraction recovery during the procedure. Considering that the structure of sodium danshensu contains multiple hydroxyls, it is easy to form intermolecular hydrogen bonds, which may affect the extraction. So acid was added to the methanol or acetonitrile to reduce the formation of hydrogen bonds between molecules. The results showed that the recovery was improved but still unacceptable; meanwhile, interference and impurities appear in the blank. Hence, conventional organic solvent deproteinization and extraction methods were evidently not suitable for sodium danshensu detection in plasma. A LLE method was then used for the plasma treatment. Several organic solvents were tested: ethyl acetate, *tert*-butyl methyl ether, a mixture of ethyl acetate and *tert*-butyl methyl ether, and hexane. The results revealed that selecting *tert*-butyl methyl ether as the extraction reagent achieved a higher extraction rate and sensitivity as well as no peak interference of the analyte. So liquid-liquid extraction with *tert*-butyl methyl ether was undertaken. In order to obtain the highest extraction rate, different acids (0.1%, 0.5%, 1%, 2%, 5% formic acid or 2 mol/L, 3 mol/L hydrochloric acid) and volumes (5 μL, 10 μL, 20 μL, 30 μL) were tested. Finally, the result was found to be satisfactory with 20 μL of 2 mol/L hydrochloric acid added in 100 μL plasma. In addition, sodium danshensu is instable at high temperature, and *tert*-butyl methyl ether can easily volatilize under nitrogen gas at 30 °C, which could guarantee the stability of the sample and improve the throughput of the experiments.

Considering the structural characteristics of the analyte, some compounds as candidates for the internal standard (IS) were evaluated in this study, including ferulic acid, protocatechuic acid, protocatechuic aldehyde, ketoprofen, and gallic acid. Finally, gallic acid was chosen as the IS because it had a more suitable retention time in the chromatographic separation as well as exhibited a stable response, effective separation, good reproducibility, and extraction efficiency.

### 2.2. Optimization of Chromatographic and MS/MS Conditions

**Figure 2:**
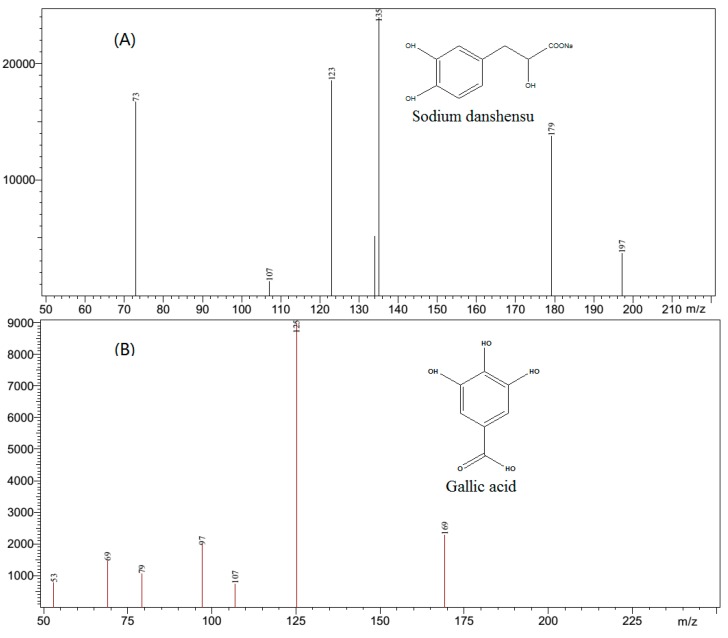
Representative product ion mass spectra of (**A**) Sodium danshensu (**B**) Gallic acid (IS).

In order to achieve a selective and sensitive method for sodium danshensu and gallic acid (IS), the MS conditions were optimized. The first step was to select a suitable ionization mode. It revealed that in the negative mode the response of the analyte was higher than that in the positive mode. In the negative ESI mode, The analyte and IS formed ions of *m*/*z* 197 [M − H]^−^ and *m*/*z* 169 [M − H]^−^, respectively. In the product ion spectra, several product ions including *m*/*z* 179, *m*/*z* 135, and *m*/*z* 123 were observed (Figure 2) for sodium danshensu and *m*/*z* 125, *m*/*z* 97, and *m*/*z* 69 were observed (Figure 2) for gallic acid. However, the ions at *m*/*z* 135 and 125 were found to be satisfactory with the acquisition of sodium danshensu and gallic acid, respectively. In order to obtain the richest relative abundance of precursor and product ions, the energy was optimized. The dwell time was also optimized for both the analyte and IS, which was crucial for the shape of the compounds. The other tandem mass spectrometer parameters including nebulizing gas flow, drying gas flow, interface voltage, interface current, DL temperature, and heat block temperature were automatically optimized.

It is known that the chromatographic conditions, especially the analytical columns, mobile phase, flow rate, and elution program were pivotal influences on the resolution, theoretical plates, signal response, symmetric peak shapes, and analysis time of the analyte and IS. Hence, several trials were performed to optimize the chromatographic conditions. Due to the high polarity of sodium danshensu, the analyte was easy to co-elute with early eluting endogenous compounds, which may have interfering peaks that affect the analytes and cause ion suppression. In order to avoid this phenomenon, the prolonged retention time is significant. In preliminary experiments, different types of columns were tested including Diamonsil, Welch, Waters, Agilent, and CNW. As a result, the CNW Athena C18-WP column (100 × 2.1 mm, 3 μm) was finally selected. The reason was that the use of this column could obtain a more suitable retention time and narrow peak width. The response signal was not satisfactory with the determination of sodium danshensu as well as the tailing that occurred when methanol-water was used as the mobile phase. Therefore, ammonium acetate and formic acid alone or with a combination in different concentrations were added. The results suggested that adding 0.2% formic acid in the water phase could solve the tailing problem and increase the ionization efficiency of the analyte. However, it does not conform to the regulation where in the negative ion mode, the addition of acid to the mobile phase can inhibit the ionization of the analyte. The possible reason was that adding acid in the mobile phase could reduce the polymerization among the analyte molecules and thereby increase the response. In addition, adding ammonium acetate in the water phase could lead to the appearance of interfering peaks. Finally, the mobile phase consisting of methanol and 0.2% (*v*/*v*) aqueous formic acid using a gradient elution was adopted.

### 2.3. Method Validation

#### 2.3.1. Selectivity

**Figure 3:**
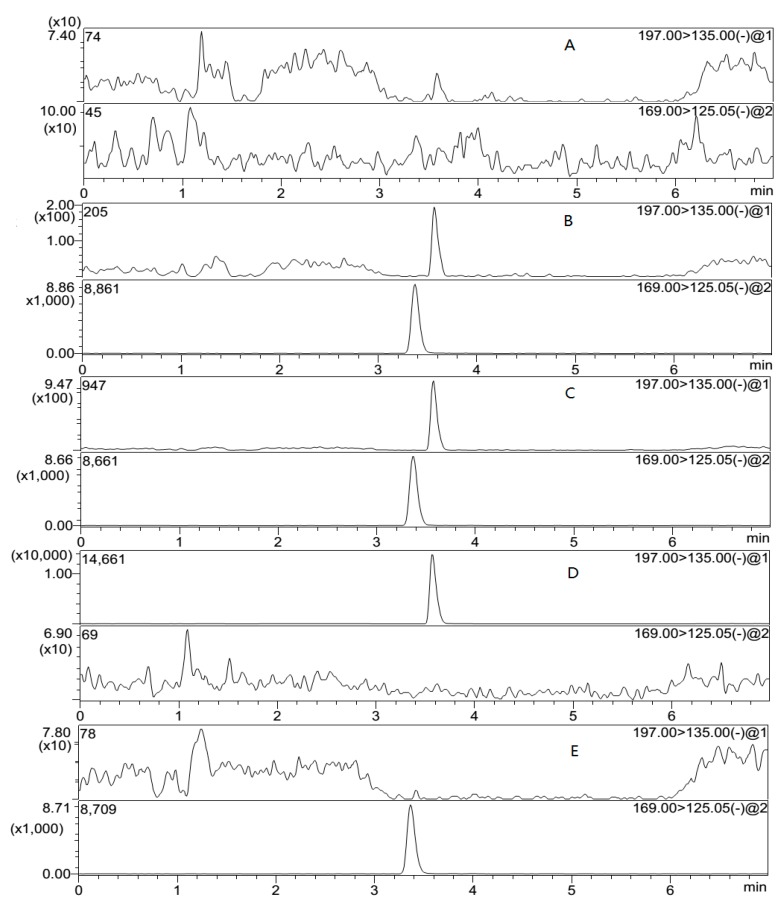
Representative MRM chromatograms of sodium danshensu and IS in beagle dog plasma samples. (**A**) A blank plasma sample; (**B**) a blank plasma sample spiked with sodium danshensu (10 ng/mL) and IS (25 ng/mL); (**C**) a plasma sample from a beagle dog 1.5 h after intravenous administration of sodium danshensu at a dose of 10 mg/kg. The assayed concentration of sodium danshensu in this sample was 930 ng/mL; (**D**) blank plasma spiked with sodium danshensu only (1000 ng/mL); (**E**) blank plasma spiked with IS only (25 ng/mL).

In order to investigate the selectivity of the method, blank plasma samples from six different sources of beagle dogs were analyzed. The retention time of sodium danshensu and IS was 3.6 and 3.4 min, respectively. The signal to noise ratio was greater than 5 at the LLOQ. Figure 3 demonstrates that no interferences were observed in the blank plasma.

In addition, the “cross-talk” of MRM channels for the analyte and IS was checked. Figure 3D,E clearly shows no MS/MS response from the analyte into the IS channel and vice versa.

#### 2.3.2. Linearity and LLOQ

The calibration curve was linear from 10 to 1000 ng/mL, which was constructed by plotting the peak area ratio of the analyte to IS (*Y*) versus the spiked plasma sodium danshensu concentration (*X*). The weighting coefficient was 1/*X*^2^. The regression equation of the analyte was *Y* = 0.0035*X* + 0.012. The linearity of the calibration curve was excellent across the calibration range, because the coefficient of determination was greater than 0.995. The lower limit of quantification (LLOQ) was used to evaluate the sensitivity of the analytical method, which required precision (RSD, %) ≤20%, and the accuracy (RE, %) ranged from −20% to 20% that certified at least five replicates. In this study, the LLOQ was 10.0 ng/mL, with an accuracy of −8.6% and a precision of 9.0% at this concentration, which was satisfactory for the analysis requirements.

#### 2.3.3. Accuracy and Precision

The intra- and inter-day precision and accuracy were assessed by measuring six replicates at the quality control (QC) concentration. The performance data for the assay are presented in Table 1. The index used to evaluate the precision was the value of relative standard deviation (RSD) and the index used to evaluate accuracy was the value of relative error (RE). The intra- and inter-day precisions were 2.3% to 9.0% and 2.1% to 6.9%, respectively. The accuracy ranged from −8.6% to 5.7%. All of the results conformed to the acceptable criteria, meaning that the method which we established was accurate, reliable, and reproducible.

#### 2.3.4. Recovery and Matrix Effect

The extraction recovery and the matrix effect for sodium danshensu and IS were assessed by measuring the QC samples. The data are shown in Table 2. The mean extraction recoveries of the analyte were all more than 80.0% at different concentration levels and the mean extraction recovery of IS was more than 90.0%. The results showed that the recoveries of sodium danshensu and IS were consistent, precise, and reproducible at the present analysis condition. Due to the mass spectrometry that was used to analyze the sample, it was necessary to determine the matrix effect. The blank plasma samples used to investigate the matrix effect in beagle dogs were obtained from six different sources. Each source of matrix was spiked with low, medium, and high concentrations of the sodium danshensu and internal standard, and all the results were within 83.8–94.9%, and the IS was 97.5%. The results indicated that no obvious matrix could influence the ionization of the analyte and IS.

#### 2.3.5. Stability

The stability of sodiusm danshensu was investigated including freeze-thaw cycle stability, ambient temperature stability, autosampler stability, and long-term stability. The samples were subjected to three cycles of freezing and thawing to assess the freeze-thaw stability and the deviation of the concentration was within the acceptable limit. Excellent stability was obtained when the samples were stored at ambient temperature for 2 h, and when stored at −80 °C for 20 days. No degradation was found after the extracted samples were kept in the autosampler for 24 h (Table 3).

#### 2.3.6. Sample Dilution

The dilution integrity experiment was performed with the aim of validating the dilution test to be carried out on higher analyte concentrations above the upper limit of quantitation (ULOQ). A set of plasma samples was prepared containing sodium danshensu at concentrations of 80,000 ng/mL, 40,000 ng/mL, 16,000 ng/mL, 8000 ng/mL, 4000 ng/mL, and 1600 ng/mL. The spiked samples were diluted with blank beagle dog plasma to generate a final concentration of 800 ng/mL. Then the samples were prepared and analyzed. The results of the sample dilution are shown in Table 4, which demonstrated that diluting high concentration samples with blank plasma did not affect the accuracy and precision of the assay.

#### 2.3.7. Auto Sampler Carryover

We evaluated the auto sampler carryover to ensure that it does not affect the accuracy and the precision of the proposed method. Sequential runs of blank and ULOQ were sequenced for analysis. The results indicated a complete carryover free auto sampler condition, and blank runs acquired post ULOQ injections did not observed any enhancement in the response. The auto sampler carryover was monitored throughout the study.

#### 2.3.8. Application to Pharmacokinetics Studies

**Figure 4:**
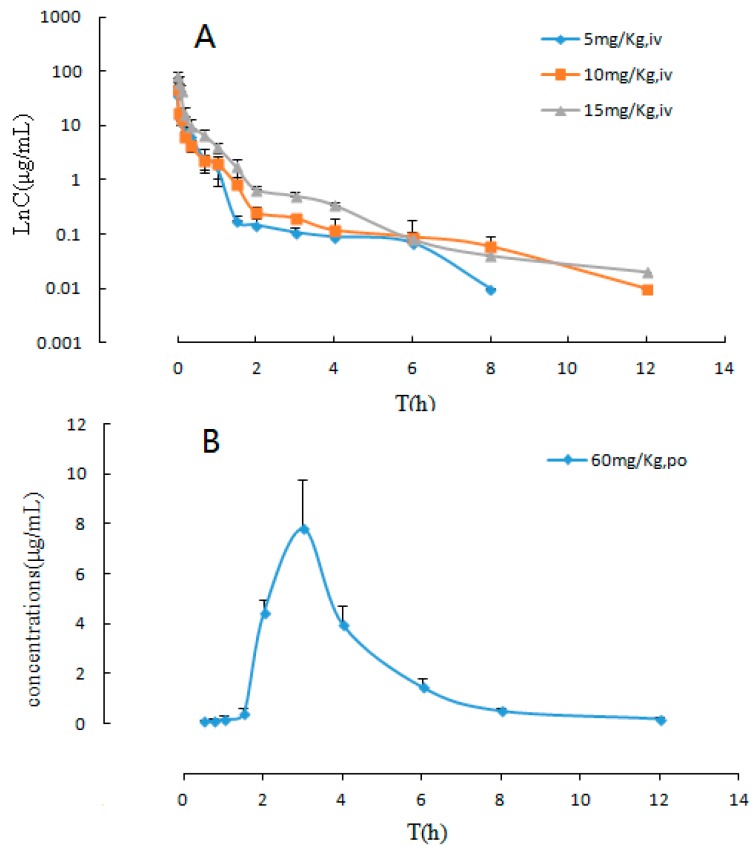
Logarithm-transformed concentration-time (LnC-T) profiles and mean plasma concentration-time profiles of sodium danshensu (each point represents mean ± SD). (**A**) LnC-T profiles after intravenous administration of sodium danshensu at doses of 5, 10, and 15 mg/kg in beagle dogs; (**B**) mean plasma concentration-time profile after oral administration of sodium danshensu at a dose of 60 mg/kg in beagle dogs.

The developed and validated LC-MS/MS method was successfully applied to the pharmacokinetic studies and bioavailability assessment of the sodium danshensu in beagle dogs after single intravenous injection doses at 5, 10, and 15 mg/kg and an oral dose at 60 mg/kg. The LnC-T profiles of sodium danshensu after intravenous administration and the mean plasma concentration time profiles of sodium danshensu after oral administration to beagle dogs are shown in Figure 4 and the main pharmacokinetic parameters are presented in Table 5. It can be seen from the data shown in Table 5 that the plasma concentration of sodium danshensu decreased quickly with an elimination half time (t_1/2_) of 2.30 ± 1.23 h, 2.32 ± 1.33 h and 1.64 ± 0.39 h upon intravenous injections of 5, 10, and 15 mg/kg, respectively. The AUC_0–t_ were 6.71 ± 0.81 μg⋅h/mL, 8.27 ± 0.38 μg⋅h/mL, and 17.75 ± 3.03 μg⋅h/mL, respectively. Our data revealed that the apparent volume of distribution (V_d_) was 6.63 ± 3.49 L for the dose of 5 mg/kg, 12.75 ± 5.50 L for the dose of 10 mg/kg, and 6.36 ± 1.48 L for the dose of 15 mg/Kg. In general, our results from the pharmacokinetic study of sodium danshensu after intravenous administration clearly indicated that sodium danshensu is distributed rapidly and widely at all dose levels. SPSS software was used to analyze the pharmacokinetic parameters of different dosages. The results of the variance analysis showed that the t_1/2_ and MRT had no significant differences among different groups (*p* > 0.05). Meanwhile there was no significant difference in AUC_0-τ_/ Dose between low dose (5 mg/kg) and high dose (15 mg/kg) (*p* > 0.05). However, the AUC_0-τ_/ Dose of middle dose (10 mg/kg) showed a significant difference with that of the low does or high dose (*p* < 0.05). Moreover, the relationship between AUC_0-τ_ and Dose was reflected in the linear correlation coefficient (*r*^2^ = 0.7636), and the AUC_0-τ_ and doses after intravenous administration tends to have a positive correlation. The C_max_ after oral administration (60 mg/kg) was found to be 7.92 ± 1.77 μg/mL, which was considerably much lower than intravenous administration. The mean absolute bioavailability (%F) of sodium danshensu was 34.8%, which was estimated via the AUC_0→__∞_ values of oral and intraveous administration.

## 3. Materials and Methods

### 3.1. Chemical and Reagents

Sodium Danshensu was provided by the consignee and its purity was approximately 98.4%. The internal standard (IS), gallic acid, was purchased from The National Institute For The Control of Pharmaceutical and Biological Products (Beijing, China) and its purity was approximately 98.0%. The methanol from Merck (Darmstadt, Germany) was of HPLC grade. *Tert*-butyl methyl ether from Anpel Laboratory Technologies (Shanghai, China) Inc. was of HPLC grade. The formic acid was purchased from Ahaqur chemicals supply (Fairfield, CA, USA). The hydrochloric acid of analytical grade was obtained from Sinopharm Chemical Reagent Co. Ltd. (Shanghai, China), Deionized water (18.2 MΏ/cm) was generated in-house using a Milli-Q System from Millipore (Bedford, MA, USA).

### 3.2. Animals and Doses

All the studies on animals were done in accordance with the Guidelines for the Care and Use of Laboratory Animals. Beagle dogs (~10 kg) were purchased from the Shanghai Slack Experimental Animal Co., Ltd. (Shanghai, China) and acclimated in the laboratory for 1 week prior to the experiments, housed in separate cages under room temperature with a 12 h light/dark cycle, and free access to standard diet and water. All the beagle dogs were fasted for 12 h before the experiments with free access to water.

For pharmacokinetics study, 24 beagle dogs were randomly divided into four groups (*n* = 6). Four different dosages (5 mg/kg, 10 mg/kg, 15 mg/kg I.V., 60 mg/kg P.O.) were designed and were assigned randomly to the four groups of beagle dogs. Blood samples (500 μL) were withdrawn from the foreleg vein into heparinized tubes in strict accordance with the time points of the protocol made in advance. Then after centrifuging at 8000 rpm for 10 min, the supernatant plasma sample was transferred into another tube and frozen at −80 °C until analysis. The time points for blood sampling were listed as follows. Pre-dose and subsequently at 0, 0.03, 0.08, 0.17, 0.33, 0.67, 1, 1.5, 2, 3, 4, 6, 8, and 12 h were used for the three intravenous injection groups, and pre-dose, 0.08, 0.17, 0.25, 0.5, 0.75, 1, 1.5, 2, 3, 4, 6, 8, and 12 h were used for the oral administration group.

### 3.3. Preparation of Calibration Standard and Quality Control Samples

The stock solutions of sodium danshensu and gallic acid were prepared in 70% methanol at a final concentration of 1 mg/mL and 0.5 mg/mL separately. All the stock solutions were stored at 4 °C in the refrigerator prior to use. Standard stock solutions were serially diluted in 70% methanol to the working solutions step by step. The calibration curve was constructed with seven concentrations (10, 20, 50, 100, 200, 500, 1000 ng/mL). Three levels (low, medium, high) of quality control samples (10 ng/mL, 20 ng/mL, and 800 ng/mL) were prepared by spiking working solutions into blank plasma.

### 3.4. Sample Preparation

For plasma sample preparation, the LLE in 96-well format plate was used. Prior to analysis, all frozen plasma samples, calibration standards, and quality control samples were thawed and allowed to equilibrate at room temperature. The samples were vortexed adequately using a vortex mixer (Beijing Targintech Co. Ltd., Beijing, China) before pipetting. Samples were prepared using LLE in 96-well deep format plate (2 mL, Corning Life Sciences—Axygen Inc., Union City, CA, USA). An automatic multichannel pipette (INTEGRA Biosciences AG, Zizers, Switzerland) was used for liquid transfer steps. Aliquots of 100 μL plasma were transferred into the 96-well deep format plate. Aliquots of 10 μL of IS solution (25 ng/mL) were added to each well except for the well designated for the double blank plasma. Then 20 μL of 2 mol/L hydrochloric acid was added to each well of the plate, and vortexed for 30 s for mixing. The mixed sample was then extracted with 1000 μL methyl *tert*-butyl ether by vortex-mixing for 3 min in a platform shaker (Grant, Cambridge, UK). After centrifugation at 12,000 rpm for 10 min, 800 μL of the upper organic layer was transferred from the original sample plates into the respective positions of the new 2.0 mL deep 96-well plates. Extracts were concentrated to dryness by an automated concentration evaporation system at 30 °C under a gentle stream of nitrogen (Turbo Vap 96, Biotage AB, Uppsala, Sweden) for 15 min. After drying, the residue was reconstituted with 100 μL of methanol–water (50:50, *v*/*v*) and was then vortexed for 3 min and centrifuged for 10 min at 12,000 rpm. A 5 μL aliquot of the supernatant was injected into the LC-MS/MS system for analysis.

### 3.5. LC-MS/MS Condition

Sample separation and determination were achieved by a Shimadzu 8040 LC-MS/MS system composed of a Shimadzu 20A solvent management system equipped with an online degasser, autosampler, column oven, and a Shimadzu tandem quadrupole mass spectrometer (Shimaszu, Kyoto, Japan).

The chromatographic separation was performed on a CNW Athena C18-WP (3 μm, 2.1 × 100 mm) column with a flow rate of 0.3 mL/min at 25 °C. The mobile phase was composed of A (water within 0.2% formic acid) and B (methanol) with a liner gradient elution of 10–60% (*v*/*v*) B at 0–0.5 min, 60% B at 0.5–4 min and 10% B at 4.01–7 min.

The ionization and detection of the analyte and IS were carried out on a triple quadrupole mass spectrometer. Quantitation was performed by negative multiple reaction monitoring (MRM) mode to monitor the parent ion→product ion (*m*/*z*) of sodium danshensu (197→135) and gallic acid (169→125), respectively. Dwell time for sodium danshensu was 350 ms and for gallic acid was 300 ms. The source-dependent parameters were set as follows: capillary voltage 3.5 kv; nebulizer gas 3 L/min; dry gas 15 L/min; DL temperature 200 °C; heat block temperature 400 °C; detector voltage 1.86 kv.

### 3.6. Method Validation

A full method validation, including selectivity, linearity, LLOQ, precision, accuracy, recovery, matrix effect, stability, sample dilution, and auto sampler carryover was conducted to evaluate the performance of the method in accordance with the guidelines published by the FDA.

#### 3.6.1. Selectivity

The selectivity of the method was tested by comparing the chromatograms of six different lots of blank plasma samples, plasma samples spiked with the sodium danshensu and gallic acid (IS), and plasma samples obtained from beagle dogs after dosing.

#### 3.6.2. Linearity and LLOQ

The calibration curve was determined by plotting the peak area ratio (*Y*) of the analyte to IS versus the nominal concentration (*X*) of the analyte with weighted (1/*X*^2^) least square linear regression. The lower limit of quantitation (LLOQ) of the assay was defined as the lowest concentration on the standard curve that can be quantitated with accuracy within 20% bias of the nominal concentration and RSD.

#### 3.6.3. Accuracy and Precision

The within- and between-run precisions were evaluated by repeating QC samples at four levels (LLOQ, low, medium, and high) with six replicate samples for each run. The accuracy was determined as RE (%) within—15–15% from the nominal values, and the precision as RSD (%) within ±15% except for LLOQ, where it should be within ±20% accuracy and not exceeding 20% of the precision.

#### 3.6.4. Matrix Effect and Recovery

The matrix effect was evaluated by the ratio of the mean peak area of the post-spiked samples to that of the neat standards at corresponding concentrations. Three QC concentrations (low, medium, high) were applied to evaluate the suppression or enhancement of the matrix. Six different lots of drug-free plasma were tested with the three QC levels and each of the QC concentrations was tested in triplicate.

The extraction recovery was calculated by comparing the mean peak responses of three QC samples of each concentration level with the mean responses of the analytes from standard solutions spiked in post-extracted blank plasma at the same concentration.

#### 3.6.5. Stability

The analyte stability determinations comprised ambient temperature stability, long-term stability, autosampler stability, and freeze-thaw cycle stability, which were evaluated by analyzing two QC levels in triplicate. The QC samples were analyzed after storage at room temperature for 2 h, at −80 °C for 20 days, and in the autosampler at room temperature for at least 24 h after extraction and after three freeze-thaw cycles, which consisted of storage at −80 °C for a minimum of 12 h followed by thawing at room temperature.

#### 3.6.6. Dilution Methods

The dilution integrity was confirmed for QC samples that exceeded the upper limit of the standard calibration curve. The results have shown that the precision and accuracy of the diluted samples were acceptable.

#### 3.6.7. Auto Sampler Carryover

The carryover effect of the autosampler was estimated by injecting a blank sample after injecting a high concentration sample or calibrating the standard sample. The carryover in the blank sample after the high concentration sample should not exceed 20% of the lower limit of the quantitation and no more than 5% of the internal standard.

### 3.7. Pharmacokinetic Calculation and Statistical Analysis

The pharmacokinetic analysis was performed using the non-compartmental model analysis. The concentration versus time data and the pharmacokinetic parameters including C_max_, t_1/2_, T_max_ (h), AUC_0-t_, AUC_0-12_, MRT, CL, and V_d_ were assessed via non-compartmental analysis using the BAPP software (version 2.0, Center of Drug Metabolism and Pharmacokinetics, China Pharmaceutical University, Nanjing, china). The absolute bioavailability (%F) of sodium danshensu was estimated via AUC_0→__∞_ values of oral and intravenous administration.

## 4. Conclusions

In this study, a new high-throughput, rapid, sensitive, and reliable LC-MS/MS analysis method to determine sodium danshensu in beagle dog plasma was successfully validated. The established assay method has been proven to be high throughput, precise, accurate, specific, reproducible, and suitable for the purpose of the pharmacokinetic study of sodium danshensu. The linearity is good over the linear range of 10–1000 ng/mL with the lower limit of quantitation of 10 ng/mL for investigating the sodium danshensu in beagle dog plasma. Unknown sample concentrations exceeding the range were diluted and re-assayed. The analytes were found to be stable in beagle dog plasma for 20 days when stored at −80 °C. The pharmacokinetic behavior of sodium danshensu tended to show linear pharmacokinetics in intravenous administration dosage ranging from 5 to 15 mg/kg and the coefficient of association (*r*^2^) of AUC_0-τ_ was 0.7636. The mean absolute bioavailability of sodium danshensu in beagle dogs was estimated as 34.8%. This method was successfully applied to the pharmacokinetic study of sodium danshensu, which will be helpful in providing a firm basis and useful information for further research into the preclinical and clinical pharmacokinetics of sodium danshensu.

## Figures and Tables

**Table 1:** Summary of accuracy and precision for the determination of sodium danshensu in beagle dog plasma.

Spiked (ng/mL)	Intra-Day (*n* = 6)			Inter-Day (*n* = 18)		
	Measured (ng/mL) (mean ± SD)	RSD (%)	RE (%)	Measured (ng/mL) (mean ± SD)	RSD (%)	RE (%)
10	9.14 ± 0.82	9.0	−8.6	9.52 ± 0.66	6.9	−4.8
20	19.78 ± 0.98	4.9	−1.1	19.88 ± 1.01	5.1	−0.6
100	105.68 ± 6.78	6.4	5.7	101.09 ± 6.57	6.5	1.1
800	804.54 ± 18.62	2.3	5.6	819.45 ± 17.68	2.1	1.8

**Table 2:** Extraction recovery and matrix effect for sodium danshensu and IS in six different sources of beagle dog plasma.

Spiked (ng/mL)	Recovery (Mean ± SD)% (*n* = 6)	RSD (%)	Matrix Effect (Mean ± SD)% (*n* = 3)	RSD (%)
20	87.4 ± 7.9	9.0	83.8 ± 4.3	5.1
100	85.2 ± 4.5	5.3	94.9 ± 6.1	6.4
800	81.3 ± 8.6	10.5	85.1 ± 1.3	1.5
25 (IS)	94.6 ± 4.1	4.3	97.5 ± 7.3	7.4

**Table 3:** Stability of sodium danshensu in beagle dog plasma (*n* = 3).

Sample Condition	Spiked (ng/mL)	Measured (ng/mL) (Mean ± SD)	RSD (%)	RE (%)
Three freeze-thaw cycles at −80 °C	20	21.45 ± 0.88	4.1	7.2
	800	798.54 ± 47.76	6.0	−0.2
Autosampler stability, 24 h	20	20.10 ± 1.22	6.1	0.5
	800	772.49 ± 29.64	3.8	−3.4
Ambient temperature stability, 2 h	20	21.00 ± 1.42	6.8	5.0
	800	771.45 ± 23.94	3.1	−3.6
Long-Term stability (20 days)	20	20.50 ± 1.13	5.5	2.5
	800	825.29 ± 55.20	6.7	3.1

**Table 4:** Summary of dilution effects of sodium danshensu in beagle dog plasma (*n* = 5).

Dilution Factor	Assayed (ng/mL) (Mean ± SD)	Reported (ng/mL) (Mean ± SD)	RSD (%)	RE (%)
2	864.91 ± 18.92	1729.81 ± 37.84	2.2	8.1
5	827.07 ± 24.66	4135.33 ± 123.29	3.0	3.4
10	755.57 ± 22.23	7555.73 ± 222.26	2.9	−5.6
20	776.61 ± 40.78	15,532.27 ± 815.66	5.3	−2.9
50	840.08 ± 53.16	42,004.00 ± 2658.02	6.3	5.0
100	803.04 ± 69.39	80,304.00 ± 6938.97	8.6	0.4

**Table 5:** Mean pharmacokinetic parameters after intravenous and oral administration of sodium danshensu in 24 beagle dogs.

Parameters	IV			PO
	5 mg/kg	10 mg/kg	15 mg/kg	60 mg/kg
C_0_ (μg/mL)	36.56 ± 3.84	46.66 ± 15.16	79.15 ± 18.92	-
C_max_ (μg/mL)	-	-	-	7.92 ± 1.77
t_1/2_ (h)	2.30 ± 1.23	2.32 ± 1.33	1.64 ± 0.39	1.84 ± 0.29
T_max_ (h)	-	-	-	2.83 ± 0.41
MRT (h)	0.88 ± 0.41	1.08 ± 0.53	0.73 ± 0.05	4.23 ± 0.20
CL (L/h)	7.37 ± 0.89	11.84 ± 0.62	8.67 ± 1.83	-
V_d_ (L)	6.63 ± 3.49	12.75 ± 5.50	6.36 ± 1.48	-
AUC_0–t_ (μg∙h∙mL^−1^)	6.71 ± 0.81	8.27 ± 0.38	17.75 ± 3.03	22.21 ± 2.48
AUC_0–∞_ (μg∙h∙mL^−1^)	6.87 ± 0.78	8.46 ± 0.43	17.79 ± 3.04	22.73 ± 2.57

## Abbreviations

HPLC-MS/MS	liquid chromatography coupled to tandem mass spectrometry
ESI	electrospray ionization
DL	desolvation line
LLOQ	lower Limit of quantitation
MRM	mean residence time
RSD	relative standard deviation
RE	relative error
MRT	mean residence time
IV	intravenous
PO	per os. [Latin], the meaning is “by mouth”
